# Patients’ Willingness and Ability to Identify and Respond to Errors in Their Personal Health Records: Mixed Methods Analysis of Cross-sectional Survey Data

**DOI:** 10.2196/37226

**Published:** 2022-07-08

**Authors:** Rachael Lear, Lisa Freise, Matthew Kybert, Ara Darzi, Ana Luisa Neves, Erik K Mayer

**Affiliations:** 1 National Institute for Health Research Imperial Patient Safety Translational Research Centre Institute of Global Health Innovation Imperial College London London United Kingdom; 2 Imperial College Healthcare National Health Service Trust London United Kingdom

**Keywords:** electronic health records, personal health records, patient participation, errors, patient safety, digital health literacy

## Abstract

**Background:**

Errors in electronic health records are known to contribute to patient safety incidents; however, systems for checking the accuracy of patient records are almost nonexistent. Personal health records (PHRs) enabling patient access to and interaction with the clinical records offer a valuable opportunity for patients to actively participate in error surveillance.

**Objective:**

This study aims to evaluate patients’ willingness and ability to identify and respond to errors in their PHRs.

**Methods:**

A cross-sectional survey was conducted using a web-based questionnaire. Patient sociodemographic data were collected, including age, sex, ethnicity, educational level, health status, geographical location, motivation to self-manage, and digital health literacy (measured using the eHealth Literacy Scale tool). Patients with experience of using the Care Information Exchange (CIE) portal, who specified both age and sex, were included in these analyses. The patients’ responses to 4 relevant survey items (closed-ended questions, some with space for free-text comments) were examined to understand their willingness and ability to identify and respond to errors in their PHRs. Multinomial logistic regression was used to identify patients’ characteristics that predict the ability to understand information in the CIE and willingness to respond to errors in their records. The framework method was used to derive themes from patients’ free-text responses.

**Results:**

Of 445 patients, 181 (40.7%) “definitely” understood the CIE information and approximately half (220/445, 49.4%) understood the CIE information “to some extent.” Patients with high digital health literacy (eHealth Literacy Scale score ≥26) were more confident in their ability to understand their records compared with patients with low digital health literacy (odds ratio [OR] 7.85, 95% CI 3.04-20.29; *P*<.001). Information-related barriers (medical terminology and lack of medical guidance or contextual information) and system-related barriers (functionality or usability and information communicated or displayed poorly) were described. Of 445 patients, 79 (17.8%) had noticed errors in their PHRs, which were related to patient demographic details, diagnoses, medical history, results, medications, letters or correspondence, and appointments. Most patients (272/445, 61.1%) wanted to be able to flag up errors to their health professionals for correction; 20.4% (91/445) of the patients were willing to correct errors themselves. Native English speakers were more likely to be willing to flag up errors to health professionals (OR 3.45, 95% CI 1.11-10.78; *P*=.03) or correct errors themselves (OR 5.65, 95% CI 1.33-24.03; *P*=.02).

**Conclusions:**

A large proportion of patients were able and willing to identify and respond to errors in their PHRs. However, some barriers persist that disproportionately affect the underserved groups. Further development of PHR systems, including incorporating channels for patient feedback on the accuracy of their records, should address the needs of nonnative English speakers and patients with lower digital health literacy.

## Introduction

### Background

Errors in electronic health records (EHRs) are not uncommon and are known to contribute to patient safety incidents [1]. Up to 60% of patient records may include inaccuracies or omissions, such as errors in patients’ diagnoses, medical history, medications, allergies, test results, procedures, contact information, and appointment details [2-6]. Early EHR systems largely reflect traditional paper–based records with records containing large amounts of unstructured free text stored in a digital format. As such, it can be time-consuming for clinicians to locate pertinent information in the patient’s record, and data entry errors can occur when health care professionals copy and paste outdated information from other parts of the record [7,8]. Computerized physician order entry and clinical decision support systems were introduced into EHRs to reduce error, and they have undoubtedly benefited clinicians and patients [9]. However, the unintended consequences of poorly designed EHR functionality and associated usability issues (eg, alert fatigue) have compounded the problem of EHR errors, creating new threats to patient safety [8,10,11]. In addition, lack of EHR interoperability can mean that important medical information entered by clinicians in different organizations is absent from the records [12]. As health care professionals use EHR data to inform clinical decision-making, errors and omissions may lead to delayed diagnoses, inappropriate treatment, and medication safety incidents [1,13,14]. Despite these known problems, there are no routine mechanisms for checking the accuracy of patients’ electronic records [4].

Whereas an EHR is a computer record that originates with and is controlled by health care professionals, a personal health record (PHR) can be generated by clinicians but is controlled by the patient. PHRs not only provide patients with access to their clinical records but also enable them to input their own data and manage who sees their records among different providers [15,16]. Recent widespread adoption of PHR systems has been driven by emphasis on transparency, an appetite for patients to become partners in their own care, and the need for health record integration not currently provided by EHR systems [15,17,18]. Evidence suggests that sharing electronic records with patients positively affects several domains of care quality, including enhanced patient engagement and involvement of informal caregivers, adherence to treatment, and timely follow-up [6,14,19-22]. Despite concerns that PHR systems could damage the patient-physician relationship if patients were to identify errors in their records, research indicates that sharing records with patients improves trust in care providers, and patients feel empowered by the opportunity to check their records for accuracy [20,22]. As the trend toward patients being able to access their electronic records accelerates through rapid digital transformation of health care systems [17], there is a valuable opportunity for patients to play a role in identifying and addressing erroneous information in the PHR. Preliminary evidence suggests that most patients can understand the information in their records to identify potential errors [2]; however, further research is needed to explore how patient involvement could be leveraged to improve the accuracy of health records. A better understanding of patients’ views around PHR error surveillance is important to uncover factors that may exacerbate the *digital divide* and linked health inequalities, such that future initiatives to involve patients in addressing PHR error are accessible to diverse patient groups.

### Objectives

The aim of this study was to evaluate patients’ willingness and ability to identify and respond to PHR errors to inform future error surveillance initiatives.

## Methods

### Study Design, Participants, and Data Collection

A cross-sectional study, using a web-based survey, explored patients’ views and experiences of using the Care Information Exchange (CIE), the largest shared PHR program in the United Kingdom. In 2018, the CIE was rolled out to the diverse 2.3 million patients treated in North West London. At the time of this survey, the CIE held patient information from both hospitals and general practitioner practices in North West London, and records from 15 hospitals outside London—in Birmingham, Bristol, Liverpool, Manchester, Scotland, and Wales [23].

The questionnaire was open for completion between July 1, 2018, and July 1, 2019. All patients registered with the CIE during the study period (N=27,411) were eligible to participate. The survey was administered via Qualtrics (a web-based survey platform). Patients registered with the CIE were invited by email to complete the questionnaire via a weblink in the portal. The email explained the purpose of the study, and informed consent was obtained. Patients had to be aged at least 18 years to register with the CIE. Not all patients registered with the CIE had used the portal; for the analyses presented, we only included respondents who indicated that they had used the CIE. We excluded patients who did not provide basic demographics regarding age and sex. Considering this population, a CI of 95%, and a margin of error of 5%, the minimum sample size to ensure representativeness was calculated as 379 respondents.

We have previously characterized individuals registered with the CIE and evaluated the differences between users and nonusers of the CIE with respect to their sociodemographic characteristics, health status, and motivation related to being involved in their own care [23]. Our findings highlight the importance of addressing educational aspects (educational level and digital literacy) to ensure equitable and sustainable portal adoption [23]. Building on this previous work, we sought to understand how portal use could be leveraged to improve patient safety and care quality. Patient portals offer users the opportunity to actively contribute to patient safety by identifying errors in PHRs and taking action to ensure that these are rectified. To conduct an initial assessment of the acceptability and feasibility of this patient safety strategy, this study aimed to evaluate the CIE users’ willingness and ability to identify and respond to errors in their EHRs.

Patients’ responses to 4 specific questions were analyzed (Textbox 1). The questions were multiple choice and closed ended, with some responses prompting the respondent to elaborate using free text.

The following sociodemographic information was used in this analysis to identify predictors of patients’ willingness and ability to identify and respond to errors in their records: age, sex, ethnicity, native language, education level, geographical location, and health status. Respondents’ level of motivation to be involved in their own care was assessed via a multiple choice question (“In general, how motivated to be involved in your healthcare are you?” Possible responses: “A little,” “A moderate amount,” “A lot,” or “Very much”). In addition, digital health literacy was assessed using the eHealth Literacy Scale (eHEALS), developed and validated by Norman and Skinner [24]. The eHEALS tool is an 8-item measure of patients’ combined knowledge, comfort, and perceived skills in finding, evaluating, and using internet health resources for health problems [24]. The 8 items are answered on a 5-point Likert scale (1-strongly disagree to 5-strongly agree); total eHEALS scores range from 8 to 40, with a higher score indicating higher digital literacy.

**Questionnaire items and format of responses.**

**Questionnaire items and responses**

1. Did you understand the information that you saw on Care Information Exchange? (*possible responses*):

- Yes, definitely
- Yes, to some extent
- No, please specify *<free-text response>*
- Not sure, please specify *<free-text response>*

2. When using Care Information Exchange, did you notice any errors in your record? (*possible responses*):

- No, I did not notice any errors
- Yes, I did notice errors. Please specify *<free-text response>*

3. If you were to see an error in your medical record, what would you like to be able to do? (*possible responses*):

- Nothing
- Flag it up to my health care professional for correction (to promote patient safety, the questionnaire included the following statement: “If you have noticed errors in your record and have not already reported them, then it is important to contact your clinical team”).
- Correct it myself
- Unsure

4. Which types of errors would you personally feel comfortable correcting? (*tick all that apply*):

- Personal information
- Medication names
- Medication doses
- Physicians’ notes
- Dates of appointments
- Diagnosis
- Other, please specify *<free-text response>*

### Analysis

To assess the effects of excluding patients with missing data regarding age and sex (78/523, 14.9%), we ran a Pearson chi-square test of homogeneity (*c*^2^) to compare the distribution of responses to survey items between the analysis sample and the missing data sample. We used descriptive statistics to quantitatively summarize respondent characteristics and users’ responses to structured survey items. Counts and proportions were calculated for categorical variables; means and SDs were calculated for continuous variables. Using the first part of the respondents’ postcodes, geographical location was categorized according to London’s official postal districts, with an additional category “other” for patients who reside outside London’s postal districts. Age was categorized into bands (<30, 31-40, 41-50, 51-65, ≥65), ethnicity was categorized into White, and all other ethnic groups were combined owing to the small numbers of patients self-identifying to individual categories of ethnic minority background.

### Multinomial Regression Analysis

We conducted multinomial regression analyses to identify the characteristics of patients that predicted (1) their ability to identify errors in their records (ie, to what extent did they understand their records?) and (2) their willingness to respond to errors in their records (ie, what action would they like to be able to take if they noticed an error in their records?) To facilitate regression analyses in the context of sparse counts (Multimedia Appendices 1 and 2), *age*, *motivation to be involved in own care*, and *digital health literacy* were treated as dichotomous variables and respondents reporting sex as “other” were excluded. After a relevant literature review, we selected an eHEALS score ≥26 to indicate higher digital health literacy and <26 to indicate lower digital health literacy [25-29]. Univariate multinomial logistic analyses were initially performed to identify potential predictors to include in the multivariable model. As suggested by Hosmer et al [30,31], we adopted the following approach to variable selection: (1) variables that demonstrated significance (*P*<.25) in the univariate analyses were entered into the preliminary multivariable model; (2) variables that were nonsignificant at *P*>.05 according to the likelihood ratio test were then removed one at a time according to the variable with the highest *P* value (backward elimination); and (3) to check for suppressor effects, variables excluded during backward selection were re-entered separately into the regression model (forward selection). Only variables that were significant at *P*<.05 (likelihood ratio test) were retained in the final multinomial regression models. Model quality comparisons were conducted using the Akaike Information Criterion [32], and the goodness of fit was assessed using Pearson chi-square statistic [31]. Effect estimates are presented as odds ratios (ORs) with 95% CIs. Analyses were conducted using Microsoft Excel (version 16.54; Microsoft Corporation) and SPSS (version 27; IBM Corp).

### Framework Analysis of Free-Text Responses

Unstructured, free-text responses were analyzed to identify emerging themes using the framework analysis method described by Ritchie and Spencer [33]. Framework analysis is a transparent and systematic approach to qualitative analysis that enables researchers to interpret data through a 5-step process: (1) familiarization with the data; (2) identification of a thematic framework (themes may be identified a priori or emerge from the data itself); (3) indexing, to explore the fit of the theoretical framework to the data; (4) charting, which involves summarizing the data into theoretical charts; and (5) mapping and interpretation, which involves checking or reviewing and synthesizing the data set as a whole [33]. In all, 2 coders (RL and LF), both experienced with framework analysis, independently coded the data. The coders discussed differences in coding to reach a consensus. Organized frameworks of inductively and deductively derived themes and subthemes were generated and applied across free-text responses. The coders worked together to complete the other stages of the analysis. Themes and subthemes were presented with verbatim quotes from the CIE users’ free-text responses.

### Ethics Approval

The study was approved as a Service Evaluation at Imperial College Healthcare National Health Service Trust (registration number: 296/2018).

### Reporting

We followed the reporting recommendations in the Strengthening the Reporting of Observational Studies in Epidemiology statement (Multimedia Appendix 3) [34].

## Results

### Respondent Characteristics

Of 1083 patients who responded to the survey, 674 (62.23%) patients indicated that they had previously used the CIE, and of these *CIE users,* 523 (77.6%) went on to complete some or all the questionnaire. The CIE users who provided basic demographic details regarding both age and sex were included in the analysis (445/523, 85.1%; +117% of the minimum target sample size); 14.9% (78/523) of respondents with missing data for either age or sex were excluded.

Of the 445 respondents, 276 (62%) were women and most were aged ≥51 years (313/445, 70.3%). Approximately two-thirds (284/445, 63.8%) of the respondents resided in London and a further 32.6% (145/445) of the respondents lived in other geographical locations in England. More than 1 in 5 (97/445, 21.8%) participants belonged to an ethnic minority group. Most were educated to a degree level or higher (292/445, 65.6%), and the mean digital literacy (eHEALS) score was 33.6 (SD 6.4). Most patients (278/445, 62.5%) considered themselves to be highly motivated in their own care. Approximately one-third (162/445, 36.4%) of the patients reported poor health status, whereas 39.8% (177/445) reported being in good health. Most patients (284/445, 63.8%) reported using the CIE at least once a month. Patient characteristics are shown in Table 1.

**Table 1:** Respondent characteristics (N=445).

Characteristics			Respondents
**Sex, n (%)**			
	Male	167 (37.5)	
	Female	276 (62)	
	Other	2 (0.4)	
	No response	N/A^a^	
**Age group (years), n (%)**			
	<30	22 (4.9)	
	31 to 40	48 (10.8)	
	41 to 50	62 (13.9)	
	51 to 64	166 (37.3)	
	>65	147 (33)	
	No response	N/A	
**Ethnicity, n (%)**			
	Ethnic minority	97 (21.8)	
	White	343 (77.1)	
	No response	5 (1.1)	
**Geographic location, n (%)**			
	London	284 (63.8)	
	Other location in England	145 (32.6)	
	No response	16 (3.6)	
**Education, n (%)**			
	Secondary school or below	118 (26.5)	
	Undergraduate or professional degree	180 (40.4)	
	Postgraduate or higher	112 (25.2)	
	No response	35 (7.9)	
**Language, n (%)**			
	English	379 (85.2)	
	Non-English	58 (13)	
	No response	8 (1.8)	
eHealth literacy (eHEALS^b^ score), mean (SD; range)			33.6 (6.4; 8-40)
**Overall health status, n (%)**			
	Good or very good	177 (39.8)	
	Neither good nor poor	106 (23.8)	
	Poor or very poor	162 (36.4)	
	No response	0 (0)	
**Motivation to be involved in own care, n (%)**			
	Not very much	6 (1.3)	
	A moderate amount	43 (9.7)	
	A lot	116 (26.1)	
	Very much	278 (62.5)	
	No response	2 (0.4)	

^a^N/A: not applicable.

^b^eHEALS: eHealth Literacy Scale.

### Patients’ Understanding of Information in the CIE

**Figure 1:**
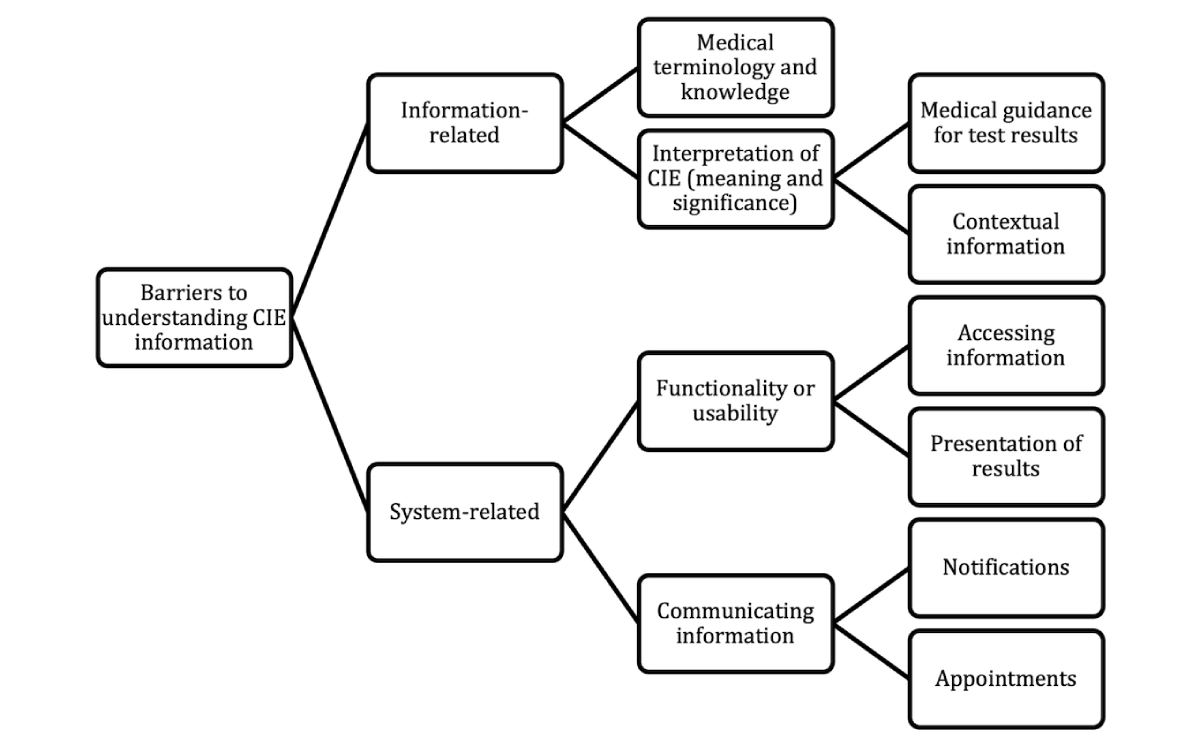
Thematic map of barriers to understanding the Care Information Exchange (CIE) information.

Of the 445 respondents, 181 (40.7%) reported that they “definitely” understood the CIE information and 220 (49.4%) reported understanding the CIE information “to some extent.” Few patients answered “No” (31/445, 7%) or “Not sure” (13/445, 2.9%); these patients were asked to elaborate on their responses and 93% (41/44) of patients provided free-text comments. Through the mapping and interpretation of coded responses, two primary categories of barriers to understanding the CIE information were identified: (1) information-related barriers (ie, users have difficulty understanding the information itself) and (2) system-related barriers (ie, problems related to the CIE system impede users’ understanding of information). These categories consist of several themes and subthemes as outlined in Figure 1.

Regarding *information-related barriers*, medical terminology or users’ lack of medical knowledge were identified as barriers to understanding the CIE information as articulated by 2 patients:

...when it is hugely medical, as one would expect for Doctors records, then I don’t always understand what I am looking at, or the relevance of its meaning.

patient ID 280

It’s great having access to blood results and radiology reports but the jargon. I don’t understand much of it.

patient ID 376

Patients often struggled to interpret the meaning or significance of information on the CIE, particularly in relation to the interpretation of test results (“Didn’t know whether a test result mattered or not” [patient ID 282]). Some patients would like more medical guidance to help with understanding the CIE information, as articulated by a patient:

There is no room or option for doctor comments so I do not understand my test results and this makes me worried when I can see them but do not understand if they are ok or not!

patient ID 412

For other patients, additional contextual information would have assisted with understanding information (“Blood tests results out of range and not knowing context” [patient ID 363]). In terms of *system-related barriers* to understanding the CIE information, patients described problems with the way in which test results were presented, including how results are displayed (“Results are virtually unreadable” [patient ID 57]) or how they are listed in the CIE:

[...] difficult to find some test results as they are listed under the medical name and the patient may not recognize the test under these names.

patient ID 195

Being able to access information was problematic for some patients; for example, a patient (patient ID 153) pointed out that that the CIE system is not accessible to screen reader users; another patient reported that “Information is too difficult to navigate when loaded separately” (patient ID 235). Some patients reported that the CIE notifications were confusing (“All these messages and they were not clear what they were for” [patient ID 428]), whereas others reported problems with how appointment information is communicated on the CIE, such as the following:

Often no detail given so it says I have an appointment but I don’t know who with or why.

patient ID 302

Overall, patients’ free-text responses provided fewer examples of system-related barriers than information-related barriers and a significant proportion of responses related to difficulties in understanding the test results.

### Patient Characteristics Associated With Understanding Information on the CIE

Patient characteristics and responses to the question “Did you understand the information you saw on CIE?” were entered into univariate and multivariable multinomial regression models to identify patient characteristics that predicted the perceived ability to understand information in the electronic record. The final multivariable multinomial regression model with 2 predictor variables (digital health literacy and motivation) predicted significantly better than the null (intercept) model (*P*<.001), and Pearson chi-square statistic suggested that the model fit the data well (*χ*^2^_2_=2.4; *P*=.30). In the final multivariable model, digital health literacy was the only variable independently associated with understanding the CIE information (Table 2). Patients with higher digital health literacy (eHEALS score ≥26) were 6 times more likely to report “definitely” understanding the CIE information, compared with patients reporting not being able to understand the CIE information (OR 6.07, 95% CI 1.70-21.57; *P*=.005). Sensitivity analyses assessing the effects of including or excluding predictor variables that demonstrated significance in the univariate analyses did not alter this result.

**Table 2:** Multinomial regression results of users’ sociodemographic characteristics and perceived level of understanding of information in the Care Information Exchange.

			Yes, to some extent vs no							Yes, definitely vs no						Yes, to some extent vs no								Yes, definitely vs no					
			Univariate odds ratio (95% CI)				*P* value			Univariate odds ratio (95% CI)			*P* value			Multivariable odds ratio (95% CI)				*P* value				Multivariable odds ratio (95% CI)					*P* value
**Sex**																													
	Female	Reference				Reference			Reference			Reference			Reference				Reference				Reference					Reference	
	Male	0.76 (0.36-1.61)				.47			0.49 (0.23-1.06)			.07			—^a^				—				—					—	
**Age (years)**																													
	≥65	Reference				Reference			Reference			Reference			Reference				Reference				Reference					Reference	
	≤64	1.33 (0.60-2.96)				.48			1.58 (0.70-3.57)			.27			N/A^b^				N/A				N/A					N/A^b^	
**Ethnicity**																													
	White	Reference				Reference			Reference			Reference			Reference				Reference				Reference					Reference	
	Ethnic minority	2.48 (0.72-8.53)				.15			3.18 (0.92-10.97)			.07			—				—				—					—	
**Native language**																													
	Non-English	Reference				Reference			Reference			Reference			Reference				Reference				Reference					Reference	
	English	0.41 (0.09-1.78)				.23			0.45 (0.10-2.00)			.29			—				—				—					—	
**Education**																													
	Secondary or below	Reference				Reference			Reference			Reference			Reference				Reference				Reference		Reference				
	Undergraduate or professional	0.47 (0.16-1.82)				.18			0.66 (0.22-1.96)			.45			—				—				—					—	
	Postgraduate or higher	0.33 (0.12-1.04)				.06			0.58 (0.18-1.82)			.35			—				—				—					—	
**Digital literacy**																													
	Lower digital health literacy	Reference				Reference			Reference			Reference			Reference				Reference				Reference					Reference	
	Higher digital health literacy	1.47 (0.51-4.18)				.48			6.23 (1.76-22.06)			.005			1.51 (0.53-4.34)				.44				6.07 (1.70-21.57)					.005	
**Health status**																													
	Neither good nor poor	Reference				Reference			Reference			Reference			Reference				Reference				Reference					Reference	
	Poor	1.48 (0.51-4.29)				.48			2.58 (0.86-7.75)			.09			N/A				N/A				N/A					N/A	
	Good	0.63 (0.25-1.57)				.32			1.29 (0.86-7.75)			.60			N/A				N/A				N/A					N/A	
**Motivation to be involved in own care**																													
	Not very much or a moderate amount	Reference				Reference			Reference			Reference			Reference				Reference				Reference					Reference	
	A lot or very much	1.08 (0.39-3.01)				.89			3.29 (1.04-10.39)			.04			0.93 (0.30-2.88)				.90				2.99 (0.85-10.61)					.09	

^a^Variable excluded from the final multivariable model using a backward elimination approach.

^b^N/A: not applicable (variable excluded from the multivariable analyses because of nonsignificance [*P*>.25] in the univariate analyses).

### Errors That Patients Have Noticed in Their Records

Nearly 1 in 5 patients (79/445, 17.8%) reported that they had noticed errors in their medical records. In all, 97% (77/79) of the patients provided further information regarding the nature of these errors. The themes and subthemes that emerged from the analysis of these free-text responses together with illustrative quotes are presented in Table 3. The most prominent theme emerging from patients’ free-text responses was *incorrect information*. This theme described 6 categories of incorrect information: patients’ details, appointments, medical history or diagnoses, measurements or results, medications, and letters or correspondence. A smaller number of respondents described information that was either incomplete or missing from the record entirely. A few patients noted instances where results or letters were missing from the record; however, most patients noted instances of missing or incomplete information related to appointments. Conflicting appointment information also emerged as a distinct theme; several users described instances of the CIE appointment information that did not match information communicated via other means (eg, through phone calls or letters). There appears to be a general lack of trust in appointment information listed on the CIE as articulated by a user:

I don’t know. Hard to confirm whether an appointment is real or a mistake as I have not received a letter or email notification to confirm appointment [sic] listed.

patient ID 303

The final theme *information belonging to a different patient* contained only 2 responses: a CIE user had noticed “another patient’s clinic letter” (patient ID 164) in their record, whereas another respondent reported that information belonging to a different patient had appeared in her midwifery notes.

**Table 3:** Types of errors patients have noticed in their records: themes, subthemes, and illustrative quotes.

Themes and subthemes			Illustrative quotes
**Incorrect information**			
	Patient details	“incorrect NHS^a^ number” (patient ID 77); “My address is wrong” (patient ID 79).	
	Appointments	“Appointment times are incorrect” (patient ID 117); “On one occasion, the system indicated that I had missed an appointment when I was definitely there” (patient ID 178).	
	Medical history or diagnoses	“Diagnosis of cancer which I do not have” (patient ID 317); “my GP^b^ said, ‘he became depressed.’ No, I didn’t” (patient ID 120).	
	Measurements or results	“A time on a test was wrong” (patient ID 304); “MRSA result appeared on my record for a test I never took” (patient ID 95).	
	Medications	“An error on the dosage of one of my medications” (patient ID 280); “I have never received ibuprofen on prescription” (patient ID 128).	
	Letters or correspondence	“Incorrect information on discharge notice from A&E^c^” (patient ID 173); “Errors in my consultants [sic] letter” (patient ID 271).	
**Missing or incomplete information**			
	Appointments	“Some appointments are not shown” (patient ID 406).	
	Measurements or results	“Blood test and urine results are missing” (patient ID 413).	
	Letters or correspondence	“2 notifications disappeared” (patient ID 264).	
Conflicting appointment information			“I am shown as having multiple appointments at different dates at the same clinic. The last time I turned up for one of these I was told there was no appointment [...] very confusing!” (patient ID 226).
Information belonging to a different patient			“Another patient’s clinic letter—major breach of confidence” (patient ID 164).

^a^NHS: National Health Service.

^b^GP: general practitioner.

^c^A&E: accident and emergency.

### Responding to Errors in the Medical Record

When asked to consider how they would like to respond to errors in their records, most patients (272/445, 61.1%) would like to flag up errors to their health professionals for correction. Although some patients (57/445, 12.8%) were unsure what action they would like to take, only a small proportion (16/445, 3.6%) said they would not take any action. Approximately one-fifth (91/445, 20.4%) of the respondents were willing to correct errors themselves. Of the 91 CIE patients who were willing to correct errors themselves, most were comfortable correcting errors in their personal information (88/91, 97%), and approximately two-thirds were willing to correct medication doses (58/91, 64%) or medication names (56/91, 62%). A smaller proportion reported willingness to correct physicians’ notes (33/91, 36%) or diagnoses (31/91, 34%).

### Patient Characteristics Associated With Willingness to Correct Errors

Patient characteristics and responses to the question, “If you were to see an error in your medical record, what would you like to be able to do?” were entered into univariate and multivariable multinomial regression models to identify patient characteristics that predicted willingness to take action to address errors in the record. The reference category for these analyses was *do nothing*. The results are presented in Table 4. The final multivariable multinomial regression model with 2 predictor variables (language and health status) predicted significantly better than the null (intercept) model (*P*=.04) and the model fit the data well (*χ*^2^_4_=6.7; *P*=.16). In multivariable analyses, users’ native language and health status predicted their willingness to take action to address errors in the record. Native English speakers were more likely to select *flag up errors to health professionals* (OR 3.45, 95% CI 1.11-10.78; *P*=.03). Native English speakers were also more likely to be willing to correct errors in their medical records themselves (OR 5.65, 95% CI 1.33-24.03; *P*=.02). Compared with patients reporting that their health was neither good nor poor (reference group), patients reporting good health status were more likely to correct errors themselves (OR 3.84, 95% CI 1.07-13.75; *P*=.04). However, there was no convincing evidence that poor health status increased the odds of being willing to correct errors (OR 3.35, 95% CI 0.89-12.61; *P*=.08). Post hoc sensitivity analyses did not affect any of these findings.

**Table 4:** Multinomial regression results of users’ sociodemographic characteristics and willingness to flag up or correct errors in the Care Information Exchange.

Characteristics		Flag it up^a^ vs do nothing		Correct it myself vs do nothing		Flag it up^a^ vs do nothing				Correct it myself vs do nothing	
		Univariate odds ratio (95% CI)	*P* value	Univariate odds ratio (95% CI)	*P* value	Multivariable odds ratio (95% CI)		*P* value	Multivariable odds ratio (95% CI)		*P* value
**Sex**											
	Female	Reference	Reference	Reference	Reference	Reference	Reference		Reference		Reference
	Male	0.71 (0.25-2.02)	.52	0.59 (0.20-1.78)	.35	N/A^b^	N/A		N/A		N/A
**Age (years)**											
	≥65	Reference	Reference	Reference	Reference	Reference	Reference		Reference		Reference
	≤64	0.81 (0.25-2.64)	.72	0.73 (0.21-2.54)	.62	N/A	N/A		N/A		N/A
**Ethnicity**											
	White	Reference	Reference	Reference	Reference	Reference	Reference		Reference		Reference
	Ethnic minority	0.48 (0.16-1.50)	.21	0.48 (0.14-1.58)	.23	—^c^	—		—		—
**Native language**											
	Non-English	Reference	Reference	Reference	Reference	Reference	Reference		Reference		Reference
	English	3.13 (1.02-9.56)	.05	3.59, (0.51-2.69)	.04	3.45 (1.11-10.78)	.03		5.65 (1.33-24.03)		.02
**Education**											
	Secondary or below	Reference	Reference	Reference	Reference	Reference	Reference		Reference		Reference
	Undergraduate or professional	1.57 (0.44-5.6)	.49	1.36 (0.36-5.21)	.65	N/A	N/A		N/A		N/A
	Postgraduate or higher	1.55 (0.36-6.75)	.56	1.80 (0.39-8.32)	.45	N/A	N/A		N/A		N/A
**Digital health literacy**											
	Low digital health literacy	Reference	Reference	Reference	Reference	Reference	Reference		Reference		Reference
	High digital health literacy	2.25 (0.47-10.83)	.31	144 (0.28-7.51)	.66	N/A	N/A		N/A		N/A
**Health status**											
	Neither good nor poor	Reference	Reference	Reference	Reference	Reference	Reference		Reference		Reference
	Poor	1.45 (0.45-4.68)	.54	3.73 (1.00-13.85)	.05	1.34 (0.41-4.41)	.63		3.35 (0.89-12.61)		.08
	Good	2.61 (0.71-9.58)	.15	5.32 (1.27-22.25)	.02	2.82 (0.76-10.54)	.12		3.84 (1.07-13.75)		.04
**Motivation to be involved in own care**											
	Not very much or a moderate amount	Reference	Reference	Reference	Reference	Reference	Reference		Reference		Reference
	A lot or very much	2.09 (0.56-7.78)	.27	2.37 (0.56-10.09)	.25	—	—		—		—

^a^Flag it up to my health care professional for correction.

^b^N/A: not applicable (variable excluded from the multivariable analyses because of nonsignificance [*P*<.25] in univariate analyses).

^c^Variable excluded from the final multivariable model using a backward elimination approach.

### Missing Data Analysis

Meaningful comparisons of sociodemographic characteristics between the analysis sample and the missing data sample were not possible because of considerable missing data in the group of 14.9% (78/523) of respondents excluded from this analysis (Multimedia Appendix 4). There were no differences in the distribution of structured questionnaire responses between the analysis sample and the missing data sample (Multimedia Appendix 5).

## Discussion

### Summary of Key Findings

A large proportion of patients could understand health information held in the CIE (fully or to some extent), and some reported that they had previously noticed errors in their records across a range of categories, including patient details, diagnoses, medical history, results, and medications. They also highlighted errors in letters or correspondence and in appointment information. Nearly two-thirds (272/445, 61.1%) of patients would like to be able to flag up PHR errors to health professionals, and some were willing to correct errors themselves; however, these patients were more likely to be native English speakers. A minority of patients with low digital health literacy had difficulty understanding information in their records. Barriers included a lack of medical knowledge or guidance, issues with portal functionality and usability, and inadequate presentation or communication of information in the portal.

### Comparison With Previous Literature

Despite policy and provider commitments enabling patient access to digital health records [17,35,36], mechanisms for systematically checking the accuracy of patient health data are almost nonexistent. Although evidence for patient involvement in improving the accuracy of their health records is limited, the findings of our study are consistent with previous research suggesting that many patients are willing and able to identify errors in their records, and their involvement may help reduce medication errors, diagnostic and treatment delays, and wasteful duplication of tests or procedures [2,4,37]. Patients can identify errors and omissions across a range of categories, including current and past diagnoses, medical or social histories, medications and allergies, procedures, test results, and appointment scheduling, with many patient-reported errors having the potential to affect care [4,7,37,38]. Evidence suggests that in many cases, patients can identify serious mistakes with clinically relevant implications that might otherwise go undetected [4,6,37-39]. This highlights the potential safety gains that can be achieved by introducing mechanisms empowering patients to engage in improving the accuracy of the personal health information. A novel finding of this study is that approximately 1 in 5 (91/445, 20.4%) patients are willing to correct errors themselves; however, no previous studies have evaluated the feasibility of this approach; key questions include which categories of errors patients could reasonably be expected to correct, as well as the safety of introducing such functionality into PHR systems.

Although patients are interested in improving the accuracy of their PHRs, certain sociodemographic factors appear to predict their readiness to participate in error surveillance. We found a significant association between native language and willingness to either correct errors or flag them up to health professionals. Similar findings from a large US study demonstrated that patients who speak a language other than English or Spanish as their primary language are less likely to report serious mistakes in electronic ambulatory visit notes [4]. We did not find any significant associations among age, sex, ethnicity, or educational level, and willingness to act on errors in the records. However, other studies have reported that patients who are male, younger, less educated, and those self-identifying as Black or African American, Asian, or from mixed ethnic backgrounds are less likely to report mistakes in their electronic records [4,38]. These findings emphasize that issues of equity must be considered when designing patient-facing error surveillance systems, such that minority groups and patients who choose not to participate in addressing error accuracy are not disadvantaged by a *digital divide* [40].

Equitable patient involvement in error surveillance is, on a basic level, contingent on all patients being able to access and understand PHR information. The characteristics of our sample are consistent with those of previous studies, reinforcing a distinct demographic profile associated with portal use, with users tending to be female, older, highly educated, with higher digital health literacy [14,22,23,41]. Previous studies have demonstrated that patients who struggle to use web-based information for health purposes are less likely to use PHR systems and that lacking these eHealth literacy skills may contribute to adverse health outcomes [23,42-44]. Our analysis reveals that patients with lower digital health literacy are also less likely to understand the information held in the PHR, which may partly explain the health disparities observed in this patient group. Consistent with previous research [2], we identified both information- and system or technology-related barriers, including a lack of support for interpreting medical information and issues with portal functionality, usability, and display of pertinent information. Patients with lower digital health literacy will continue to be underserved by digital transformation unless the barriers impeding access to, and understanding of, PHR information are addressed [40].

Interestingly, digital health literacy, as measured by the eHEALS score, did not predict patients’ willingness to correct errors in their PHRs. This finding could be explained by the properties of the eHEALS scale, which was developed according to the definition of eHealth literacy as “the ability to seek, find, understand, and appraise health information from electronic sources and apply the knowledge gained to addressing or solving a health problem” [45]. This definition was based on the first generation of static, read-only, health information technology (Web 1.0), which has led to the eHEALS being criticized as not being sufficiently comprehensive to measure the skills required to interact with and contribute to dynamic web-based health tools [46]. As suggested by one of the original authors of eHEALS, the content of the scale should be updated to reflect the modern complex, dynamic, and social nature of the web [47].

### Policy Implications and Future Research

Establishing a feedback mechanism that encourages patients to identify and respond to PHR errors is aligned with the National Health Service Patient Safety Strategy, which sets out expectations for the involvement of patients, families, caregivers, and other lay people in providing safer care, allowing patients to move from being passive recipients of care to *vigilant stakeholders* [48,49]. However, patient-facing error surveillance systems would need to align with the diverse needs, preferences, and capabilities of patients, while also providing frontline staff and health care organizations with the opportunity to learn from errors [4,20,38]. Policy makers agree that more needs to be done to avoid marginalizing specific patient groups when implementing new digital health technologies [50]. An important first step is to ensure that the accessibility, functionality, and usability of patient portals meet the needs of all patients, including those who have difficulty interacting with digital content, nonnative English speakers, and other traditionally underserved groups. Ongoing development of PHR systems, incorporating channels for patient feedback on EHR accuracy, should integrate user-centered principles such that equal access and use can be achieved for all patients while also respecting individual choice and level of engagement [20,40]. Future research should seek to understand which categories of errors patients can reliably correct and examine the feasibility and safety of patient-facing error surveillance systems while continuing to address the impact of the digital divide and associated health inequalities.

### Strengths and Limitations

A major strength of this study lies in the application of a mixed methods approach, which led to a comprehensive understanding of users’ willingness and ability to be involved in PHR error surveillance in a diverse patient population. We ensured rigor and transparency in our qualitative analyses by applying the framework method to derive insights from patients’ free-text responses [51]. We collected and analyzed a comprehensive set of patient characteristics, allowing us to explore classic demographic factors (age, sex, ethnicity, and educational level) in combination with important additional variables: health status, motivation to be involved in one’s own care, and digital health literacy. This study included patients treated in North West London, which may limit the generalizability of our findings. However, of note, around one-third (145/445, 32.6%) of the survey respondents lived outside London, in other locations across England. Future studies should use geographical data to inform the relationship between social or material deprivation and patient portal engagement. This web-based survey study examined the views of patients who have access to and experience of using an electronic PHR, and the findings therefore reflect the views of a self-selected group of digitally empowered patients. Although we achieved a minimum sample size to ensure representativeness and adequate statistical power, the survey response rate was low. CIs for the association between patients’ sociodemographic characteristics and the outcomes of interest were wide, indicating a potentially broader range of plausible predictors than could be detected in our study. Despite these limitations, our finding that digital health literacy predicts patients’ understanding of portal information resonates with the current evidence and interest in reducing health inequalities.

### Conclusions

Our findings demonstrate that patients are both able and willing to identify and respond to errors in their PHRs, although some barriers to understanding information in PHR systems persist and may disproportionately affect patients with lower digital health literacy. Further development of PHR systems, incorporating channels for patient feedback on the accuracy of their records, should integrate user-centered design principles such that equal access and engagement in mitigating errors in the record is possible for all patients.

## Appendix

Multimedia Appendix 1 Cross-tabulation of patients’ sociodemographic characteristics and their understanding of the Care Information Exchange information.

Multimedia Appendix 2 Cross-tabulation of patients’ sociodemographic characteristics and their preferences for responding to errors in their records.

Multimedia Appendix 3 The Strengthening the Reporting of Observational Studies in Epidemiology statement—checklist of items that should be addressed in reports of observational studies.

Multimedia Appendix 4 Sociodemographic characteristics of patients in the missing data and analysis samples.

Multimedia Appendix 5 Missing data analysis for structured questionnaire items.

## Abbreviations

CIE: Care Information Exchange
eHEALS: eHealth Literacy Scale
EHR: electronic health record
NIHR: National Institute for Health Research
OR: odds ratio
PHR: personal health record
