# Supplementary material for: Patients’ Willingness and Ability to Identify and Respond to Errors in Their Personal Health Records: Mixed Methods Analysis of Cross-sectional Survey Data
Source: J Med Internet Res. 2022 Jul 8;24(7):e37226. doi: 10.2196/37226 (PMC9308067; doi:10.2196/37226)
Supplement: Multimedia Appendix 1 [file jmir_v24i7e37226_app1.docx]

Table S1. Cross-tabulation of patients’ sociodemographic characteristics and their understanding of information in CIE

| **Did you understand the information you saw on CIE?** | | | | | | | |
| --- | --- | --- | --- | --- | --- | --- | --- |
|  |  | No | Not sure | Yes, to some extent | Yes, definitely |  |  |
|  |  | n (%) | n (%) | n (%) | n (%) | χ ^2^ | *P* |
| **Sex** |  |  |  |  |  | 22.17 | .001 |
|  | Female | 16  (5.8) | 8  (2.9) | 128  (46.4) | 124  (44.9) |  |  |
|  | Male | 15  (9.0) | 4  (2.4) | 91  (54.5) | 57  (34.1) |  |  |
|  | Other | 0  (0.0) | 1  (50.0) | 1  (50.0) | 0  (0.0) |  |  |
| **Age** |  |  |  |  |  | 9.84 | .63 |
|  | ≤30 | 3  (13.6) | 0  (0.0) | 9  (40.9) | 10  (45.5) |  |  |
|  | 31-40 | 4  (8.3) | 3  (6.3) | 19  (39.6) | 22  (45.8) |  |  |
|  | 41-50 | 3  (4.8) | 2  (3.2) | 36  (58.1) | 21  (33.9) |  |  |
|  | 51-64 | 9  (4.8) | 4  (3.2) | 80  (58.1) | 73  (33.9) |  |  |
|  | ≥65 | 12  (8.2) | 4  (2.7) | 76  (51.7) | 55  (37.4) |  |  |
| **Ethnicity** |  |  |  |  |  | 4.08 | .25 |
|  | Ethnic minority | 3  (3.1) | 3  (3.1) | 46  (47.4) | 45  (46.4) |  |  |
|  | White | 28  (8.2) | 10  (2.9) | 173  (50.4) | 132  (38.5) |  |  |
| **Education** |  |  |  |  |  | 11.53 | .07 |
|  | Secondary school or below | 5  (8.1) | 0  (0.0) | 69  (58.5) | 44  (37.3) |  |  |
|  | Undergraduate/ professional degree | 13  (7.2) | 7  (3.9) | 85  (47.2) | 75  (41.7) |  |  |
|  | Postgraduate or higher | 10  (8.9) | 5  (4.5) | 46  (41.1) | 51  (45.5) |  |  |
| **Language** |  |  |  |  |  | 1.91 | .59 |
|  | Non-english | 2  (3.4) | 1  (1.7) | 31  (53.4) | 24  (41.4) |  |  |
|  | English | 29  (7.7) | 12  (3.2) | 182  (48.0) | 156  (41.2) |  |  |
| **Digital Literacy** |  |  |  |  |  | 40.99 | <.001 |
|  | High digital literacy | 17  (5.2) | 6  (1.8) | 142  (43.0) | 165  (50.0) |  |  |
|  | Low digital literacy | 11  (11.6) | 4  (4.2) | 67  (70.5) | 13  (13.7) |  |  |
| **Health status** |  |  |  |  |  | 12.21 | .06 |
|  | Good | 16  (9.0) | 3  (1.7) | 78  (44.1) | 80  (45.2) |  |  |
|  | Neutral | 8  (7.5) | 5  (4.7) | 62  (58.5) | 31  (29.2) |  |  |
|  | Poor | 7  (4.3) | 5  (3.1) | 80  (49.4) | 70  (43.2) |  |  |
| **Motivation to be involved in own care** |  |  |  |  |  | 20.47 | .02 |
|  | Very much | 13  (4.7) | 8  (2.9) | 126  (45.3) | 131  (47.1) |  |  |
|  | A lot | 13  (11.2) | 4  (3.4) | 59  (50.9) | 40  (34.5) |  |  |
|  | A moderate amount | 5  (11.6) | 1  (2.3) | 29  (67.4) | 8  (18.6) |  |  |
|  | Not very much | 0  (0.0) | 0  (0.0) | 4  (66.7) | 2  (33.3) |  |  |
