# Supplementary material for: Patients’ Willingness and Ability to Identify and Respond to Errors in Their Personal Health Records: Mixed Methods Analysis of Cross-sectional Survey Data
Source: J Med Internet Res. 2022 Jul 8;24(7):e37226. doi: 10.2196/37226 (PMC9308067; doi:10.2196/37226)
Supplement: Multimedia Appendix 2 [file jmir_v24i7e37226_app2.docx]

Table S1. Cross-tabulation of patients’ sociodemographic characteristics and their preferences for responding to errors in their records

| **If you were to see an error in your medical records, what would you like to be able to do?** | | | | | | | |
| --- | --- | --- | --- | --- | --- | --- | --- |
|  |  | Do nothing | Unsure | Flag it up to health professional | Correct it myself |  |  |
|  |  | n (%) | n (%) | n (%) | n (%) | χ^2^ | *P* |
| **Sex** |  |  |  |  |  | 13.55 | .04 |
|  | Female | 8  (3.0) | 36  (13.3) | 167  (61.6) | 60  (22.1) |  |  |
|  | Male | 7  (4.3) | 21 (12.9) | 104  (63.8) | 31  (19.0) |  |  |
|  | Other | 1  (50.0) | 0  (0.0) | 1  (50.0) | 0  (0.0) |  |  |
| **Age** |  |  |  |  |  | 13.24 | .35 |
|  | ≤30 | 2  (9.5) | 3  (14.3) | 7  (33.3) | 9  (42.9) |  |  |
|  | 31-40 | 3  (6.3) | 6  (12.5) | 30  (62.5) | 9  (18.8) |  |  |
|  | 41-50 | 2  (3.3) | 8  (13.3) | 40  (66.7) | 10  (16.7) |  |  |
|  | 51-64 | 5  (3.0) | 18  (11.0) | 107  (65.2) | 34  (20.7) |  |  |
|  | ≥65 | 4  (2.8) | 22  (15.4) | 88  (61.5) | 29  (20.3) |  |  |
| **Ethnicity** |  |  |  |  |  | 1.73 | .63 |
|  | Ethnic minority | 5  (5.3) | 13  (13.8) | 57  60.6 | 19  (20.2) |  |  |
|  | White | 9  (2.7) | 44  (13.1) | 212  (62.9) | 91  (21.4) |  |  |
| **Education** |  |  |  |  |  | 1.56 | .96 |
|  | Secondary school or below | 5  (4.3) | 13  (11.1) | 74  (63.2) | 25  (21.4) |  |  |
|  | Undergraduate/ professional degree | 5  (2.9) | 20  (11.4) | 116  (66.3) | 34  (19.4) |  |  |
|  | Postgraduate or higher | 3  (2.7) | 12  (10.8) | 69  (62.2) | 27  (24.3) |  |  |
| **Language** |  |  |  |  |  | 4.96 | .18 |
|  | Non-english | 5  (8.8) | 8  (14.0) | 34  (59.6) | 10  (17.5) |  |  |
|  | English | 11  (3.0) | 47  (12.7) | 234  (63.1) | 79  (21.3) |  |  |
| **Digital Literacy** |  |  |  |  |  | 5.11 | .16 |
|  | High digital literacy | 11  (3.4) | 36  (11.1) | 204  (63.2) | 72  (22.3) |  |  |
|  | Low digital literacy | 3  (3.2) | 18  (19.4) | 57  (61.3) | 12  (16.1) |  |  |
| **Health status** |  |  |  |  |  | 17.03 | .009 |
|  | Good | 4  (2.3) | 20  (11.4) | 113  (64.2) | 39  (22.2) |  |  |
|  | Neutral | 6  (5.8) | 22  (21.2) | 65  (62.5) | 11  (10.6) |  |  |
|  | Poor | 6  (3.8) | 15  (9.6) | 94  (60.3) | 41  (26.3) |  |  |
| **Motivation to be involved in own care** |  |  |  |  |  | 13.23 | .15 |
|  | Very much | 8  (2.9) | 36  (13.2) | 169  (61.9) | 60  (22.0) |  |  |
|  | A lot | 5  (4.5) | 10  (8.9) | 75  (67.0) | 22  (19.6) |  |  |
|  | A moderate amount | 3  (7.0) | 8  (18.6) | 24  (55.8) | 8  (18.6) |  |  |
|  | Not very much | 0  (0.0) | 3  (50.0) | 3  (50.0) | 0  (0.0) |  |  |
