# Supplementary material for: Patients’ Willingness and Ability to Identify and Respond to Errors in Their Personal Health Records: Mixed Methods Analysis of Cross-sectional Survey Data
Source: J Med Internet Res. 2022 Jul 8;24(7):e37226. doi: 10.2196/37226 (PMC9308067; doi:10.2196/37226)
Supplement: Multimedia Appendix 3 [file jmir_v24i7e37226_app3.docx]

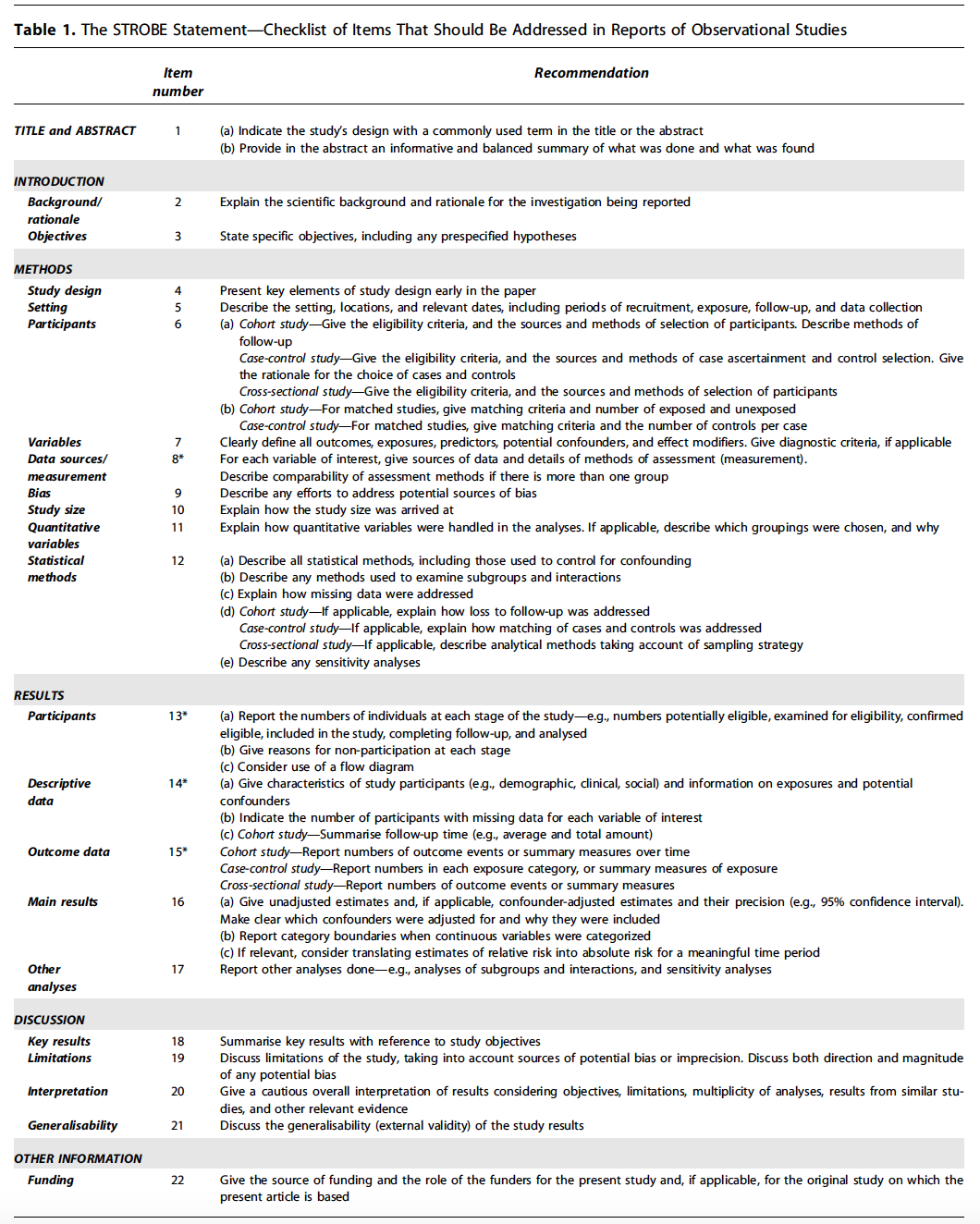


Source:

von Elm E, Altman DG, Egger M, Pocock SJ, Gøtzsche PC, Vandenbroucke JP; STROBE Initiative. The Strengthening the Reporting of Observational Studies in Epidemiology (STROBE) statement: guidelines for reporting observational studies. *PLoS Med.* 2007 Oct 16; 4(10):e296. PMID: 17941714
