# Supplementary material for: Patients’ Willingness and Ability to Identify and Respond to Errors in Their Personal Health Records: Mixed Methods Analysis of Cross-sectional Survey Data
Source: J Med Internet Res. 2022 Jul 8;24(7):e37226. doi: 10.2196/37226 (PMC9308067; doi:10.2196/37226)
Supplement: Multimedia Appendix 4 [file jmir_v24i7e37226_app4.docx]

Table S1: Sociodemographic characteristics of patients in the missing data sample and in the analysis sample

|  | Missing data sample^(a)^  (n=78) | Analysis sample  (n=445) |
| --- | --- | --- |
| **Sex** | n (%) | n (%) |
| Male | 0 (0.0) | 167 (37.5) |
| Female | 7 (9.0) | 276 (62.0) |
| Other | 0 (0.0) | 2 (0.4) |
| No response | 71 (91.0) | 0 (0.0) |
| **Age Group** | | |
| <30 | 0 (0.0) | 22 (4.9) |
| 31-40 | 0 (0.0) | 48 (10.8) |
| 41-50 | 0 (0.0) | 62 (13.9) |
| 51-64 | 0 (0.0) | 166 (37.3) |
| >65 | 2 (2.6) | 147 (33.0) |
| No response | 76 (97.4) | 0 (0.0) |
| **Ethnicity** | | |
| Ethnic minority | 5 (6.4) | 97 (21.8) |
| White | 5 (6.4) | 343 (77.1) |
| No response | 68 (87.2) | 5 (1.1) |
| Language | | |
| English | 12 (15.4) | 379 (85.2) |
| Non-English | 2 (2.6) | 58 (13.0) |
| No response | 64 (82.1) | 8 (1.8) |
| **Geographic location** | | |
| London | 7 (9.0) | 284 (63.8) |
| Other location in England | 2 (2.6) | 145 (32.6) |
| No response | 69 (88.5) | 16 (3.6) |
| **Education** | | |
| Secondary school or below | 3 (3.8) | 118 (26.5) |
| Undergraduate/professional degree | 4 (5.1) | 180 (40.4) |
| Postgraduate or higher | 5 (6.4) | 112 (25.2) |
| No response | 66 (84.6) | 35 (7.9) |
| **eHealth Literacy (eHEALS score)** | | |
| Higher eHealth literacy ≥30 | 8 (10.3) | 329 (73.9) |
| Lower eHealth literacy ≤29 | 5 (6.4) | 97 (21.8) |
| No response | 65 (83.3) | 19 (4.3) |
| **Overall Health Status** | | |
| Good or very good | 9 (11.5) | 177 (39.8) |
| Neither good nor poor | 2 (2.6) | 106 (23.8) |
| Poor or very poor | 4 (5.1) | 162 (36.4) |
| No response | 63 (80.8) | 0 (0) |
| **Motivation to be involved in own care** | | |
| Very much/a lot | 11 (14.1) | 394 (88.5) |
| Not very much/a moderate amount | 4 (5.1) | 49 (11.0) |
| No response | 63 (80.8) | 2 (0.4) |

^(a)^Respondents who did not report age and/or sex were excluded from the analysis.
