# Supplementary material for: Patients’ Willingness and Ability to Identify and Respond to Errors in Their Personal Health Records: Mixed Methods Analysis of Cross-sectional Survey Data
Source: J Med Internet Res. 2022 Jul 8;24(7):e37226. doi: 10.2196/37226 (PMC9308067; doi:10.2196/37226)
Supplement: Multimedia Appendix 5 [file jmir_v24i7e37226_app5.docx]

Table S1: Missing data analysis for structured questionnaire items

|  | Missing data^(a)^  sample | Analysis sample |  |  |
| --- | --- | --- | --- | --- |
|  | n (%) | n (%) | χ^2^ | *P* |
| **Did you understand the information you saw on CIE?** | | | .94 | .82 |
| No | 6 (7.7) | 31 (7.0) |  |  |
| Not sure | 1 (1.3) | 13 (2.9) |  |  |
| Yes, to some extent | 41 (52.6) | 219 (49.3) |  |  |
| Yes, definitely | 30 (38.5) | 181 (40.8) |  |  |
| No response | 0 | 1 |  |  |
| **When using CIE, did you notice any errors in your record?** | | | 1.84 | .18 |
| No, I did not | 68 (88.3) | 360 (82.0) |  |  |
| Yes, I did notice errors | 9 (11.7) | 79 (18.0) |  |  |
| No response | 1 | 6 |  |  |
| **If you were to see an error in your medical record, what would you like to be able to do?** | | | 5.13 | .16 |
| Correct it myself | 8 (10.5) | 91 (20.9) |  |  |
| Flag it up to my healthcare professionals | 55 (72.4) | 271 (62.3) |  |  |
| Unsure | 9 (11.8) | 57 (13.1) |  |  |
| Nothing | 4 (5.3) | 16 (3.7) |  |  |
| No response | 2 | 10 |  |  |

^(a)^Respondents who did not report age and/or sex were excluded from the analysis.
